# Supplementary material for: Time‑resolved multi-omic analysis of paclitaxel exposure in human iPSC‑derived sensory neurons unveils mechanisms of chemotherapy‑induced peripheral neuropathy
Source: Cell Death Dis. 2026 Feb 10;17(1):211. doi: 10.1038/s41419-026-08445-2 (PMC12921266; doi:10.1038/s41419-026-08445-2)
Supplement: Supplementary file 15 — Dataset 1. [file 41419_2026_8445_MOESM15_ESM.zip › CDD_Open_Data/04_RNA_Seq/Timeline_panel_plots_etc_2023-01-23_1609.zip!/Panel_Plots_etc/Evidence_Plots/72h/evidence_plot_standard contrasts_msigdb_go_bp_72h_PTX_vs_DMSO_ID0_M13808_GOBP_REGULATION_OF_CALCIUM_ION_DEPENDENT_EXOCYTOSIS.pdf]

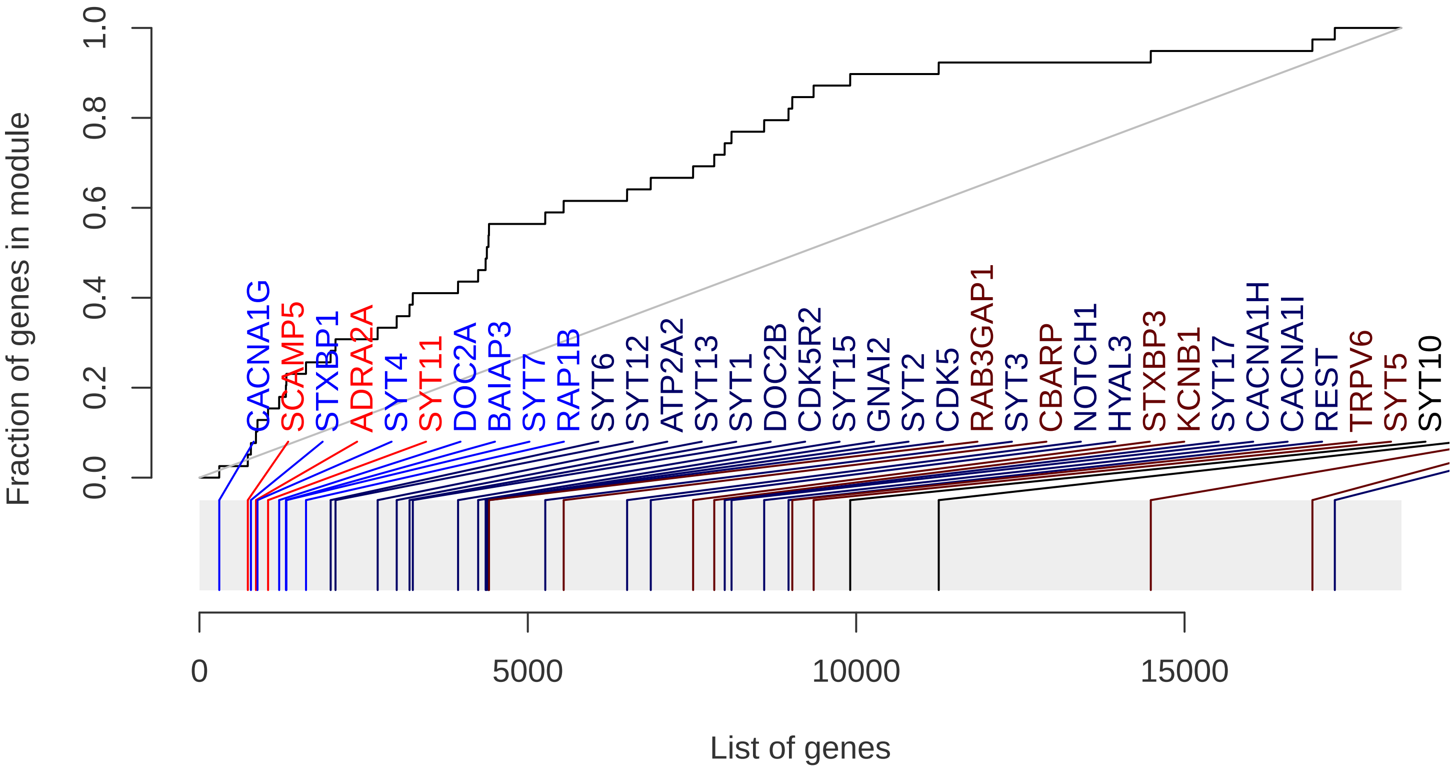

Fraction of genes in module
0.0
0.2
0.4
0.6
0.8
1.0
CACNA1G
SCAMP5
STXBP1
ADRA2A
SYT4
SYT11
DOC2A
BAIAP3
SYT7
RAP1B
SYT6
SYT12
ATP2A2
SYT13
SYT1
DOC2B
CDK5R2
SYT15
GNAI2
SYT2
CDK5
RAB3GAP1
SYT3
CBARP
NOTCH1
HYAL3
STXBP3
KCNB1
SYT17
CACNA1H
CACNA1I
REST
TRPV6
SYT5
SYT10
0
5000
10000
15000
List of genes
